# Identifying missing links in the conceptualization of financial toxicity: a qualitative study

**DOI:** 10.1007/s00520-021-06643-6

**Published:** 2021-10-30

**Authors:** Sara L. Lueckmann, Nadine Schumann, Christoph Kowalski, Matthias Richter

**Affiliations:** 1 Institute of Medical Sociology, Medical Faculty, Martin Luther University Halle-Wittenberg, Magdeburger Str. 8, 06112 Halle (Saale), Germany; 2 German Cancer Society, Department for Health Services Research, Kuno-Fischer-Straße 8, 14057, Berlin, Germany

**Keywords:** Qualitative research, Neoplasm, Financial distress, Health, Psycho-oncology, Cancer survivors, Patient-reported outcome

## Abstract

**Purpose:**

Financial toxicity can have a major impact on the quality of life of cancer survivors but lacks conceptual clarity and understanding of the interrelationships of the various aspects that constitute financial toxicity. This study aims to extract major drivers and mediators along the pathway from cancer-related costs to subjective financial distress from the patients’ experiences to establish a better understanding of financial toxicity as a patient-reported outcome.

**Methods:**

Qualitative semistructured interviews with 39 cancer patients were conducted in Germany and addressed patient experiences with cancer-related financial burden and distress in a country with a statutory health care system. Transcripts were analyzed using content analysis.

**Results:**

Several aspects of financial burden need to be considered to understand financial toxicity. The assessment of the ability to make ends meet now or in the future and the subjective evaluation of financial adjustments—namely, the burden of applied financial adjustments and the availability of financial adjustment options—mediate the connection between higher costs and subjective financial distress. Moreover, bureaucracy can influence financial distress through a feeling of helplessness during interactions with authorities because of high effort, non-traceable decisions, or one’s own lack of knowledge.

**Conclusion:**

We identified four factors that mediate the impact of higher costs on financial distress that should be addressed in further studies and targeted by changes in policies and support measures. Financial toxicity is more complex than previously thought and should be conceptualized and understood more comprehensively in measurements, including the subjective assessment of available adjustment options and perceived burden of financial adjustments.

## Introduction

The costs due to a cancer diagnosis can have a major impact on treatment and patient outcomes. Over the last decade, the financial consequences of cancer diagnosis and their adverse long-term effects have received increasing attention, often referred to as financial toxicity (FT) [1]. Carrera et al. showed that cancer patients respond to higher medical costs and income loss with changes in consumption, which can have an impact on distress, indebtedness, and non-compliance with medical treatment, thereby influencing quality of life and health [1]. An association between financial distress and depression and anxiety is evident up to 10 years after diagnosis [2].

Even though a unified understanding and measurement of FT is lacking [3], there is consensus on the clear distinction between objective financial burden attributable to cancer and related subjective financial distress [1, 4, 5]. Objective financial burden is often measured as the increase in individual costs. Measurement of subjective financial distress often includes material, psychosocial, and behavioural aspects [6]. To date, our understanding of FT is mainly based on quantitative studies analyzing the prevalence of or risk factors for any measure (objective or subjective) or the associations between measures and patient outcomes. The present work defines FT as the mechanism by which the objective burden of higher costs becomes subjective financial distress. To develop interventions to reduce patients’ subjective financial distress and improve their quality of life, a better understanding of the relationship between higher costs and financial distress is needed [7]. Initial studies show that out-of-pocket costs and lost income are associated with financial distress [8–10]. However, this does not fully describe the context, as individuals in the same objective financial situation may perceive different levels of subjective financial distress.

It is likely that subjective financial distress does not derive directly from the existence and extent of higher costs [11]. First, risk factors for objective or subjective measures are not the same [12]. Second, there is only a low level of consistency and no significant association between patients’ objective and subjective financial burdens [13, 14].

To date, little is known about *how* objective financial burden becomes subjective financial distress. Carrera et al. point out that changes in consumption and other, potentially maladaptive, coping mechanisms frame this association [1]. Some studies suggest that making financial adjustments, e.g. delaying or avoiding care, applying for financial assistance, using savings, or reducing spending, is associated with financial distress [15, 16]. Factors such as having a mortgage/personal loan, poor workability, changes to employment, higher household bills, having dependents, or financial distress before diagnosis have been shown to be associated with subjective financial distress after diagnosis [8, 17] and have thus been put forth as possible explanatory factors of the association between higher costs and financial distress. However, these findings were often based on cross-sectional data, and causal mechanisms of FT remain mainly unclear [18].

Qualitative studies that analyze patients’ experiences of cancer-related costs and their subjective perceptions of financial distress and its emergence can shed light on relevant mediators and help to gain greater conceptual clarity of FT [19]. Generally, financial consequences from cancer are more pronounced in countries with high out-of-pocket costs for health care. The German health care system is a multipayer health care system with a combination of compulsory statutory (approximately 90% of the population) and private insurance. Most health care costs are covered by statutory health insurance with only low out-of-pocket costs that are capped at 2% (1% for chronically ill persons) of the gross annual household income upon request. During sickness, income loss is typically mitigated by sickness benefits, which cover approximately 70% of an individual’s regularly relevant total gross income for up to 78 weeks upon application. Subsequently, benefits covered by the social security system can be claimed, such as unemployment benefits or reduced earning capacity pensions. Therefore, studies in countries with universal health care and overall lower financial impact of diseases can help to better explain the emergence of subjective financial distress in cancer patients. Therefore, we aim to analyze major drivers and mediators along the pathway from cancer-related objective financial burden to subjective financial distress to establish missing links in the construct of FT from the patients’ experiences.

## Methods

We chose a qualitative design with cancer survivors to capture potential mediators of the relationship between objective financial burden and subjective financial distress.

### Recruitment of participants

Between May 2017 and April 2018, we gradually recruited participants, conducted interviews, and analyzed data. We reached potential participants in diverse ways, mainly through contacts at hospitals and resident specialists and through flyers. In total, 58 participants consented to taking part in an interview on the financial consequences of their cancer diagnoses. Initially, the inclusion criteria for this study were as follows: (i) a diagnosis of a first breast, prostate, lung, or colorectal cancer; (ii) > 30 years of age; and (iii) completion of acute treatment for cancer within the last 5 years. At the beginning of the sampling process, we included every suitable patient. During purposive sampling, maximal variation was considered in terms of age, gender, socioeconomic status, family status, extent of financial impact, and financial distress, and of the 58 patients recruited, only 39 were interviewed with the potential to expand the developed theory until sampling saturation was reached. For the purposes of contrasting and reaching saturation, we had to broaden the inclusion criteria and included some patients within the 39 with other tumour entities, cancer recurrence, a second diagnosis of cancer, or patients who were in long-term therapy. Eligible participants were informed about the study, and in-depth semistructured interviews were conducted after patients provided written informed consent.

### Conduction of the interviews

A semistructured interview guideline was used, including main questions related to changes in financial situation, consequences of financial changes for participants’ lives, and handling of financial changes. Depending on each participant’s choice, the interviews took place at the scientific institute, at the participant’s home, or during aftercare appointments. Two female research associates (SLL, an economist, and NS, a sociologist) with years of experience in qualitative health research conducted the interviews face to face. Field notes were recorded after each interview. The interviews normally included only the participant and one interviewer, but in seven interviews, family members were present at the request of the participant. The interviews lasted between 23 and 160 min, with a mean length of 68 min. All but one interview was audio-recorded with the interviewees’ permission.

### Analysis

**Fig. 1:**
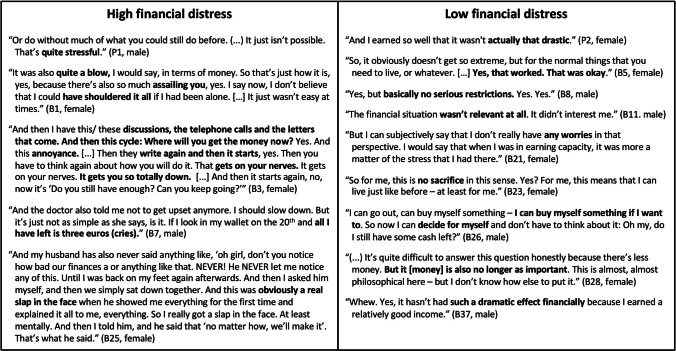
Examples of expressions that led to the categorization into high or low subjective financial distress

The transcripts of the interview records were analyzed using pseudonyms according to qualitative content analysis [20]. All interviews were conducted and analyzed in the German language. For the quotations and interview guide presented in this manuscript, we conducted a double-blind translation from German to English. Initially, six interviews were double-coded “data-driven” by trained coders from the qualitative working group at the scientific institute and were used to develop an initial code structure with categories. Subsequently, four interviews were double-coded by SLL and NS to enhance the coding tree. Either SLL or NS coded the remaining transcripts, and the respective other researcher checked the coding; discrepancies were discussed. The patients were categorized as having a high objective financial burden if the reported total costs, considering all financial changes, exceeded 10% of the net household equivalent income in the short term, or 5% in the long term (for at least 2 years). The remaining patients were categorized as having a low objective financial burden. Patients’ subjective financial distress was categorized as high or low by SLL and NS based on subjective expressions, and categorizations were discussed afterwards to reach a consensus. The classification was based on two independent assessments of the interview, but exemplary quotes that helped in the decision-making can be found in Fig. 1. We compared and contrasted the patients’ experiences related to the objective financial situation and subjective financial distress. MAXQDA 12.0 software was used to facilitate coding and comparison of the groups.

## Results

**Fig. 2:**
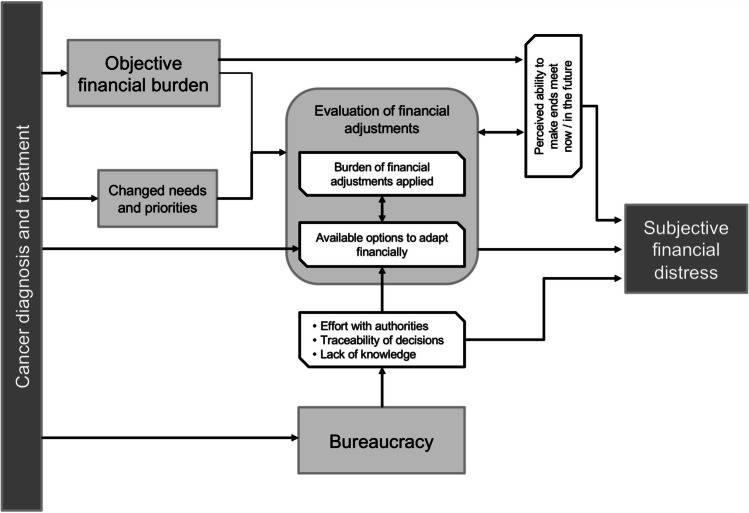
Model of financial toxicity based on the patients’ experiences

The patients’ characteristics are displayed in Table 1. Three out of 18 patients with low objective financial burden experienced subjective financial distress, and of those with high objective financial burden, approximately half experienced subjective financial distress. Comparing 12 financially distressed and 22 low-stress cancer patients (the remaining 5 patients could not be clearly assigned to either group), we derived four main distressing factors that patients can feel exposed to and that may cause subjective financial distress (see Fig. 2). These factors are related to the *evaluation of financial adjustment options*, the *burden related to applied adjustments*, the *perceived ability to make ends meet*, and *bureaucracy*; these factors are described in more detail below, and illustrative quotes showing exemplary contents can be found in Table 2. These factors are embedded in the context of the disease and cancer-related higher costs that interact with disease-related changes in needs and priorities, e.g. ascribing less value to consumption compared to health or more value to treating oneself.

**Table 1:** Patient characteristics (39 patients)

Characteristic	Description	Sample distribution
Age	Mean age (range)	58.7 years (40–86)
Gender	Female	18
	Male	21
Tumour entities*^1^	Breast	11
	Colon	9
	Lung	7
	Prostate	7
	Other	5
Employment^1^	Occupied	23
	Self-employed	5
	Old-age pensioners	5
	Unemployed	3
	Reduced earnings capacity pensioners	2
Household and dependents^2^	Partner, parents, older children	21
	Single	10
	Families (living with children)	6
	Single parent	2
Monthly household net equivalent income^2^	< 1200 €	13
	1200 to 1800 €	12
	> 1800 €	13
	Missing	1
Relation of objective financial burden with subjective financial distress	High objective financial burden	21
	*Subjectively financially distressed*	*9*
	*Not subjectively financially distressed*	*7*
	*Not clearly assigned*	*5*
	Low objective financial burden	18
	*Subjectively financially distressed*	*3*
	*Not subjectively financially distressed*	*15*

^*^With the aim of achieving maximal variation in individual courses of disease and career paths, we included patients with tumour entities other than breast, prostate, lung, and colon cancer and patients with a second cancer diagnosis or recurrence whose first cancer diagnosis was made more than 5 years ago

^1^(At the time of getting) the most recent cancer diagnosis

^2^At the time of the interview

**Table 2:** Exemplary quotes for the four main factors that were found to mediate the impact of higher costs on financial distress

**Evaluation of financial adjustment options**
“Insanely on the psyche. The psyche is the worse part because you just don’t know how—if you don’t have anyone to financially support you, this is an insanely big burden because you don’t know/ So you buy less. After all, you still have to pay for everything somewhere with the small budget that you have—pay for insurance policies, pay for electricity, pay for water and so on. All of this still continues. And you have to have enough left to pay for the ongoing costs every month. Even though you may have it, but then you have nothing left to live on.” (B18, male)
“So I asked them [the siblings] at some point whether they could give me a bit of [financial] support. Yes. This was immediately blocked by all sides, and I didn’t like that. Well. Because I always say, oh my, this can happen to anyone. It can happen to anyone to suddenly need help right away. Yes. I don’t think that’s nice. Yes. I don’t think that’s nice at all.” (B1, female)
“But I don’t believe that I’ll ever find something here—let’s say that I could earn a little extra money. (…) After all, I think once a year had passed, one of the [company’s] employees was here for the re-integration measures. And I had to also/ went through a number of positions where I could start working here. So you could say that everything was irrelevant. It was too warm at one of them, and I had to go through a temporary work agency. The other one had work that was too heavy and dusty. After all, the doctor had also included this for me [in the certificate of capacity for work]. And now finding something later that’s somewhere close to here, that’s nearby since we’re also dependent on the car—I probably won’t be able to get anything anywhere here.” (P1, male)
**Burden of applied financial adjustments**
“Nothing happens in my life anymore. Nothing at all. […] We used to go away a lot just for the weekend. (Voice breaks) Right? None is this is possible now. We went out for coffee once in a while. These are things that I can’t afford anymore. (Crying)” (B19, female)
“Because that’s what I actually didn’t want at all—that I now have to go begging my father since he always needs his money himself. And so I said as soon as I can do something, then it will be paid back again.” (P1, male)
“So back when we, the time that I mentioned earlier, just had food for poor people to eat. Even a packet of soup—a lazy woman’s soup—never. We don’t have anything like that anymore. Because we didn’t know about something like that, such as packaged or tinned food. We didn’t have things like that before. But we had to have it now and then during those times. So that my two at least had something to eat. And I kept myself going with tea and rusks. There wasn’t much that I could do anyway. But at least my two had something to eat. And now I’m doing well again. We have an income again. And that means that I can make goulash once in a while on the weekend. And that we are eating quite differently again.” (B25, female)
**Perceived ability to make ends meet**
“Well, the fact that you have to consider whether you can still afford the rent every month or not—and how far you have to overdraw your bank account—is really terrible. Losing your flat or no longer having electricity or whatever, this is the worst thing.” (B17, female)
“And the worst thing is the financial burden, because you don’t know how and what will happen. All it takes is for the car to break down with a bill of 500 or 600 euros, let’s say, which I can’t afford. Then I have to get rid of the car.” (P1, male)
“Well, it’s actually the thought that you have to manage with the little money that you have and experience this poverty among the elderly that they talk about on television. That’s actually it. So I thought that I could save a bit, a little bit more money and set it aside. Then you have to experience this poverty in old age. (…) I have to get through this now.” (B10, male)
**Bureaucracy—effort, traceability, and knowledge**
“That’s also what I said—that somehow someone should tell you about what you can do, where you can even get money at all and how you can maybe still get into a better situation. Nothing. Nothing at all. The pension fund, they just—as I already said, it was mostly in writing—turned me down. Yes, turned me down. So it’s just a good thing that I had an advisor, that someone who works for the pension fund and who explained things to me. ‘That’s unbelievable’, is what he said. ‘Halle is really bad. Saxony-Anhalt is really bad. Saxony is bad’, he said. ‘Over in West Germany, you would have already long had a pension. Even without a rejection or objection and without anything’. Well, then I wrote to the pension office once again, which is what they wanted. And I wrote about how I was doing and explained the situation. And also once again summarised the situation of my illness, everything that I have. And after the second year, the pension came forever—until 2024 or something like that.” (B7, male)
“And that’s why I hope that when the discharge report gets here, at least the EU pension office will say: ‘Ms. Krüger, then we will at least give you the EU pension for now’. And hopefully a bit retroactively. Because I had already had been applying for it since March of this year. And it has been rejected over and over again. She [social worker] had tried everything—really everything. I have all of the correspondence, which Ms. [social worker] has always also sent to me. But she says that these people are really (…), they have no conscience. They don’t know how someone down here in [place] who has now also got cancer is doing here if they have never seen the person. I can’t evaluate a person if I’m sitting at a desk in Berlin and say that this person just needs to go to rehab now and then he’ll come back healthy and can really get going again.” (B19, female)
“There are the applications. You always think that you’re not completely stupid, but sometimes you really don’t know what they want from you. Yes, and then, um, then you first have to go back to them. What did you ask? And then, yes, then you run back to your employer. And then you run to the health insurance company. Yes.” (B4, female)
“We assumed that the money would keep coming. There was no indication that it would stop and only continue once you have started (…) and a few other little things like that. […] You already have a lot to do in dealing with yourself, but now you can first gather everything that you still need—and that’s a pity.” (B1, female)

### Evaluation of financial adjustment options

Patients evaluated financial adjustments with regard to their availability. Financial distress arose from insufficient adaptive options, i.e. when patients felt they could not reduce expenditures or saw no way to increase resources through, in particular:

i. money or financial help from third parties, including being rejected when asking for help or being met with a lack of understanding regarding financial problems;
ii. earning extra money, which was not possible due to the regional labour market situations or their own physical constitutions;
iii. not having savings to fall back on.

The perceived burden through the evaluation of options to adjust the financial situation through reducing expenditures or increasing resources was influenced by the extent of current non-reducible costs, existence of savings, quality of the social network, and job opportunities. High costs that influenced the emergence of financial distress included paying loan instalments, needing a car, and having dependents. Feeling ashamed of one’s financial situation can result in not asking relatives, friends, or authorities for help, and this can influence the evaluation of financial adjustment options. Having savings, few obligations, a social and accommodating employer, and the offer of altruistic help from others improved the evaluation of available adjustment options and reduced the emergence of distress. Contact with authorities could also help with determining available financial adjustments.

### Burden of applied financial adjustments

Financial adjustments were evaluated with regard to the associated burden due to their implementation. Financial distress occurred when patients had to accept necessary expenditure cutbacks that made them feel they could no longer afford anything or when they tried to increase their resources by:

i. consuming savings or incurring bank debts that changed their expected future financial situation,
ii. getting help from third parties, which made them feel dependent, ashamed for needing help, or stressed while asking for or considering help, especially when being held responsible for their financial situations.

The perceived burden when reducing expenditures was influenced by the assessment of the necessities of consumption against the backdrop of one’s own needs. The main question was how easy is it for someone to spend less money?

### Perceived ability to make ends meet

The assessment of the ability to make ends meet now and/or in the future is influenced by the increased costs and the evaluation of the aforementioned available and applied financial adjustments. Perceived financial distress in the current situation arose from the feeling of constantly having to deal with the financial situation caused by income decline, higher direct medical costs, waiting times on cash disbursements, the unpredictability of the timing of payments, and the exclusion of remedies/aids for refund. Anticipated financial distress in the future arose from worrying about the ability to deal with future unexpected events and costs or fearing old-age poverty. Overall, the evaluation of the ability to make ends meet and the financial adjustments influenced each other, e.g. the feeling of being forced to make burdensome financial adjustments to make ends meet, or fearing the inability to make ends meet in the future because of consuming savings now.

The perceived burden in evaluating one’s ability to make ends meet was influenced by financial education, financial behaviour, and perceived ability to get by with little money. Money management became challenging, especially as falling ill came unexpectedly and the related financial decline was unpredictable. An already strained financial situation at the time of the cancer diagnosis exacerbated the perceived inability to make ends meet.

### Bureaucracy—effort, traceability, and knowledge

Patients could feel helpless in dealing with bureaucracy. Bureaucracy affected the patients and their experience of financial distress when they felt helpless in dealing with authorities and agencies, including banks and (statutory) insurance agencies. The perceived financial distress of bureaucracy occurred due to the following:

i. time-consuming and complex processes when dealing with bureaucracy,
ii. incomprehensible decisions by authorities,
iii. confrontation with one’s own lack of knowledge about rules and regulations when dealing with authorities.

Financial distress arose when patients felt overwhelmed with applications and perceived their abilities to defend themselves as limited. They felt abandoned by the authorities because of a lack of satisfactory counselling on costs and claims or because of the great effort needed to contact counselling, even though they went to the cancer counselling services for help in dealing with bureaucracy or their financial situations.

The perceived burden of experiences with bureaucracy was influenced by knowledge and mental capabilities to handle information and paperwork. In addition, good experiences with social security institutions, prompt approvals, timely authorizations, help with inquiries about one’s own rights and unsolved questions, and sufficient information about one’s rights mitigated the feeling of helplessness in dealing with bureaucracy.

## Discussion

To our knowledge, this is the first study establishing missing links in the construct of FT by analyzing patients’ subjective experiences along the pathway from cancer-related costs to subjective financial distress. We found that subjective financial distress arises from the interplay of multiple distress-causing factors, particularly:

- a lack of options to adapt financially;
- feeling burdened by the financial adjustments that have to be made, such as reducing spending and consumption, borrowing money, or using savings;
- a perceived inability to make ends meet now and/or in the future; and
- effortful contact with authorities and being confronted with non-traceable decisions and one’s own lack of knowledge.

Generally, our findings are in line with previous findings suggesting that several aspects of financial burden need to be taken into account to understand FT [6]. Other recent studies also highlight the importance of taking into account the perceived ability to make ends meet and the burden related to the application of financial adjustments when measuring financial distress [1, 5, 6, 21–24].

Additionally, we show that the subjective assessment of financial adjustments is a key factor for the emergence of subjective financial distress and is more important than the existence and extent of higher costs or applied financial adjustments. Special emphasis is placed on the individually perceived burden of applied financial adjustments and the evaluation of the availability of financial adjustment options. This can also explain contradictory findings with respect to help from third persons—money and support from family and friends or having a partner can help deal with the costs and mitigate distress [8, 25], or it can increase financial distress [23, 26]. We propose that this depends on the evaluation of the burden of accepting help from others.

To date, the assessment of the availability of financial adjustment options has not been considered in the literature. Nevertheless, having savings, employment opportunities, and high fixed expenses are significant drivers of financial distress [8, 21, 22, 27, 28]. These factors could be interpreted as reflecting the availability of options to adjust to the financial situation. Carrera et al. proposed that the application of potential maladaptive financial adjustments is the link between objective costs and financial distress [1]. We illustrate that the patient’s subjective evaluation of available options and burden of applied financial adjustments determines whether financial adjustments become maladaptive or are experienced as relieving, which is an important missing link in the construct of FT.

We assume that having savings at diagnosis strongly influences assessment of the financial situation. This is also reflected in the fact that increased FT is associated with having savings covering less than one month, even after adjusting for income [29]. Savings and household net worth are associated with higher financial literacy [30]. Financial literacy empowers people to manage their finances, which could be related to a strong competence in identifying different options for financial adjustments, not perceiving them as a burden, a greater knowledge of rules and regulations, and the confidence to find solutions to make ends meet; together, these are the factors that influence the emergence of financial distress. This implies that higher financial literacy might be helpful to empower patients in dealing with the financial burden of their illness [30, 31]. One study showed that cancer patients rated a financial literacy course as helpful in navigating the cost of cancer care, and the most important content they wanted to be included in the course was employment issues, barriers to talking about costs, and resources for financial support [21].

Bureaucracy is an additional missing link in the construct of FT. Difficult contact with authorities, a lack of knowledge about procedures, and incomprehensible decisions mediate the relationship between objective costs and financial distress. This has thus far rarely been addressed in studies, although as a partial aspect of bureaucracy, transparency of expected costs has been identified as a key component of financial distress, and knowledge of what to expect was therefore put forth as an important factor to reduce distress [10, 21, 28]. Patient counselling and assistance programmes have been found to mitigate financial distress [21]. Consulting services can help patients cope with difficult bureaucracy processes. In the authors’ view, however, it would be equally advisable, in addition to expanding counselling services, to make an effort to simplify and automate procedures and requests for (cancer) patients so that applications are less time-consuming, decisions about benefits are more transparent and comprehensible, and regulations are generally less complex and easier to understand, so that patients are not overwhelmed when dealing with authorities and bureaucracy.

Existing instruments measuring subjective financial distress often include only the evaluation of the psychosocial responses in a single question on financial difficulties, or they ask about the existence of passive financial resources, support-seeking, changes in financial spending, or coping with care or with ones’ lifestyle [6]. Based on our results, we recommend that the patients’ circumstances and subjective evaluation of the situation be considered in future questionnaires. Items should not be too specific and cover the patients’ individual assessment of financial adjustments, including burden and availability, current and future perspective on the ability to make ends meet, and experiences with bureaucracy, which we found to be important missing links in the construct of FT.

### Limitations

Although we conducted 39 interviews and reached saturation, the generalizability of the findings to all patients with cancer may be limited, e.g. to young cancer patients or families of children with cancer or patients from other countries or regions. Especially for long-term cancer survivors, other factors might be of relevance that lead to long-term financial distress. Due to the social security system with statutory health insurance in Germany, direct medical costs are lower than in other countries and probably less important for the emergence of financial stress. Nevertheless, the four identified main factors that mediate financial distress should be of international interest. It should be highlighted that this qualitative study is limited in scope, but further research should explore the validity of this model in quantitative studies with different samples and in other contexts.

## Conclusion

In this study, we confirm that subjective financial distress can arise from higher costs and other distress-causing factors, such as maladaptive financial adjustments and an inability to make ends meet, indicating that measurement should be multifactorial. Nevertheless, we find that despite asking about the existence of financial adjustments in questionnaires, the subjective assessment of available options to adjust to the financial situation and perceived burden of the financial adjustments that have to be made is relevant for evaluating subjective financial distress. In addition, the burden of bureaucracy—due to effort, lack of traceability, or knowledge deficits—must be considered if subjective financial stress is to be analyzed comprehensively. In the overall context of FT, patients experiencing several of the four identified factors might feel that the financial side effects of cancer—higher costs, changed priorities and needs, or bureaucracy—dominate one’s life.

## Data Availability

Participants of this study were guaranteed that only the study research team would have access to the interviews and transcripts; thus, data are not available for sharing.
